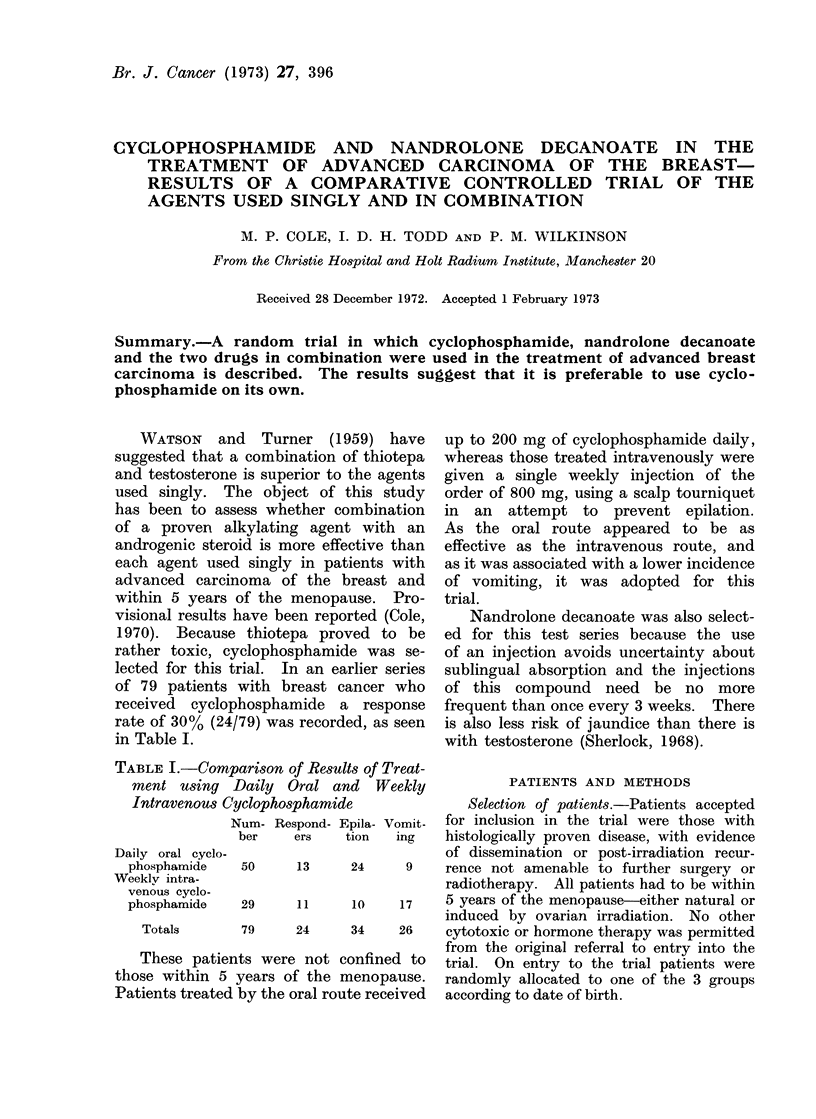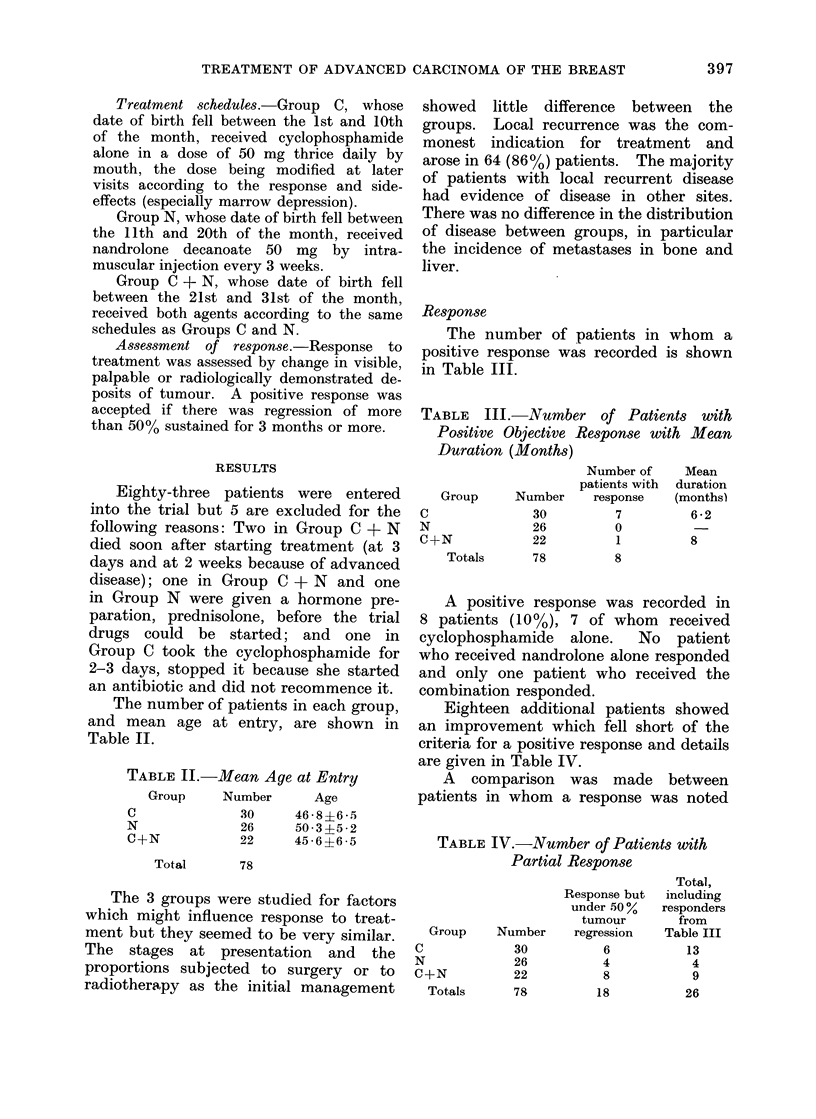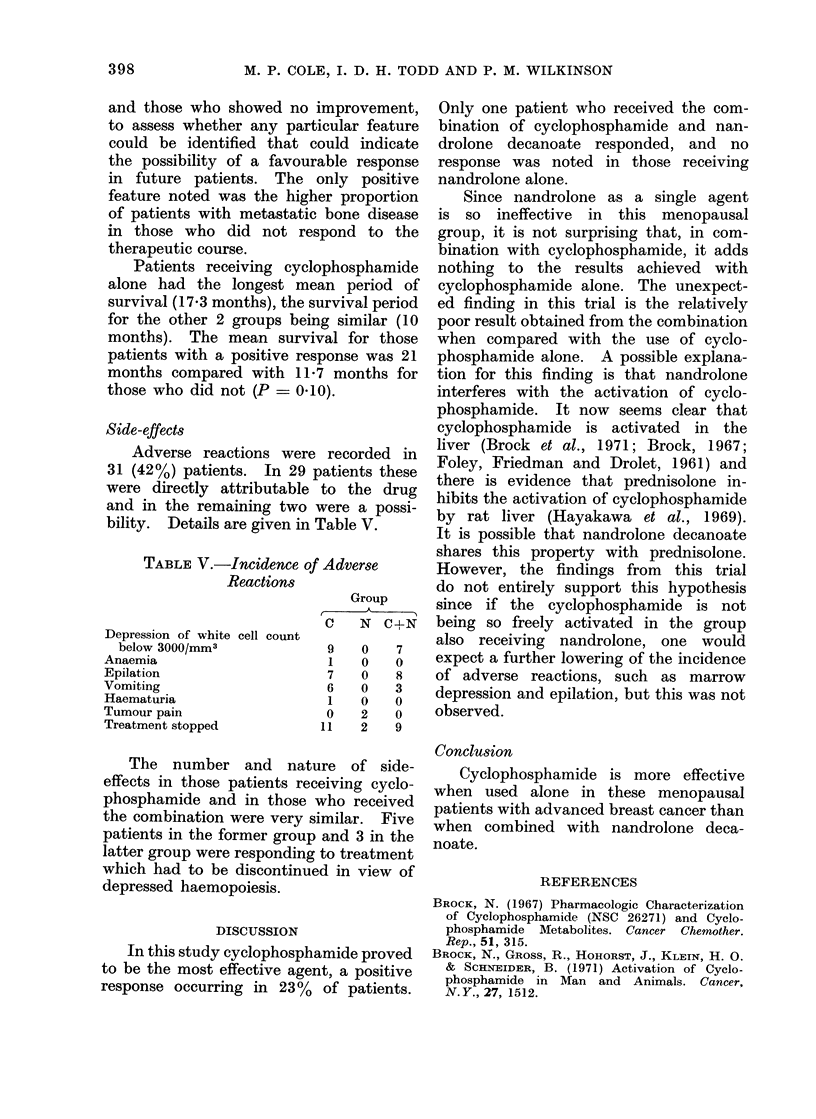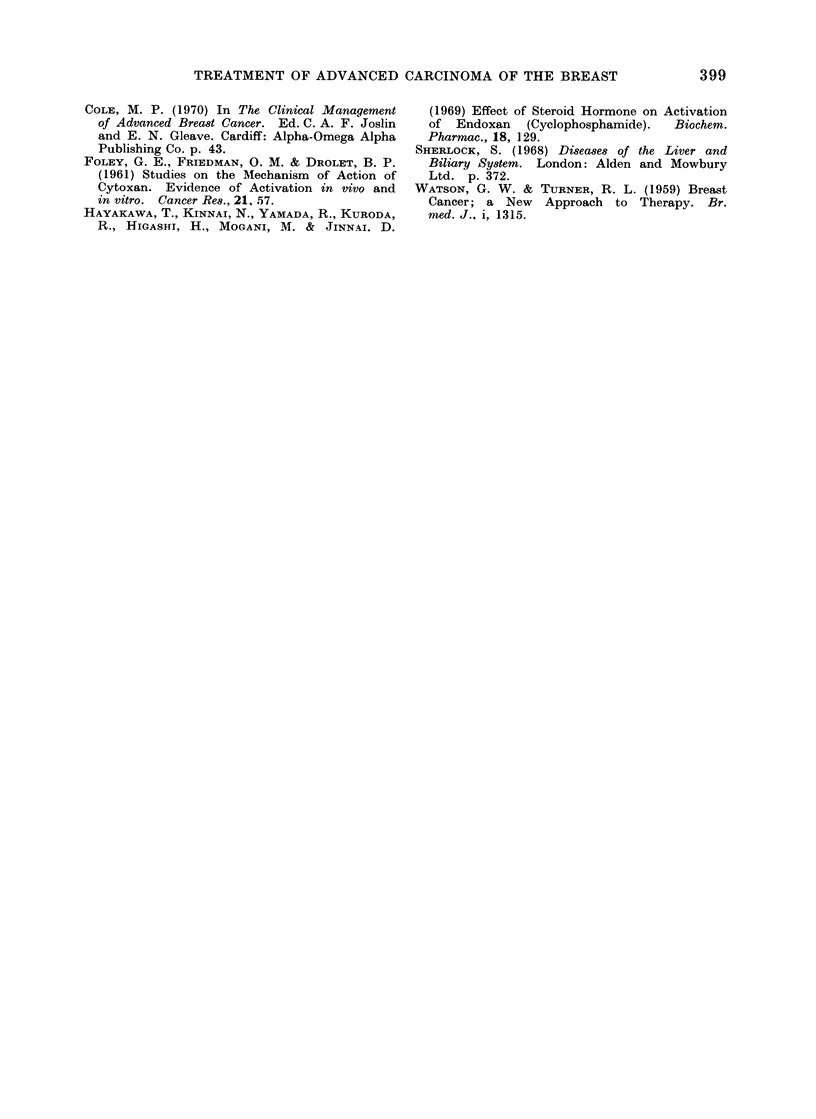# Cyclophosphamide and Nandrolone Decanoate in the Treatment of Advanced Carcinoma of the Breast—Results of a Comparative Controlled Trial of the Agents Used Singly and in Combination

**DOI:** 10.1038/bjc.1973.47

**Published:** 1973-05

**Authors:** M. P. Cole, I. D. H. Todd, P. M. Wilkinson

## Abstract

A random trial in which cyclophosphamide, nandrolone decanoate and the two drugs in combination were used in the treatment of advanced breast carcinoma is described. The results suggest that it is preferable to use cyclophosphamide on its own.


*Br. J. Cancer* (1973) **27**, 396

### CYCLOPHOSPHAMIDE AND NANDROLONE DECANOATE IN THE TREATMENT OF ADVANCED CARCINOMA OF THE BREAST—RESULTS OF A COMPARATIVE CONTROLLED TRIAL OF THE AGENTS USED SINGLY AND IN COMBINATION

M. P. COLE, I. D. H. TODD AND P. M. WILKINSON

*From the Christie Hospital and Holt Radium Institute, Manchester* 20

Received 28 December 1972. Accepted 1 February 1973

**Summary.—A random trial in which cyclophosphamide, nandrolone decanoate and the two drugs in combination were used in the treatment of advanced breast carcinoma is described. The results suggest that it is preferable to use cyclophosphamide on its own.**

WATSON and Turner (1959) have suggested that a combination of thiotepa and testosterone is superior to the agents used singly. The object of this study has been to assess whether combination of a proven alkylating agent with an androgenic steroid is more effective than each agent used singly in patients with advanced carcinoma of the breast and within 5 years of the menopause. Provisional results have been reported (Cole, 1970). Because thiotepa proved to be rather toxic, cyclophosphamide was selected for this trial. In an earlier series of 79 patients with breast cancer who received cyclophosphamide a response rate of 30% (24/79) was recorded, as seen in Table I.

TABLE I.—*Comparison of Results of Treatment using Daily Oral and Weekly Intravenous Cyclophosphamide*

| | Number | Responders | Epilation | Vomiting |
|---|---|---|---|---|
| Daily oral cyclophosphamide | 50 | 13 | 24 | 9 |
| Weekly intravenous cyclophosphamide | 29 | 11 | 10 | 17 |
| Totals | 79 | 24 | 34 | 26 |

These patients were not confined to those within 5 years of the menopause. Patients treated by the oral route received up to 200 mg of cyclophosphamide daily, whereas those treated intravenously were given a single weekly injection of the order of 800 mg, using a scalp tourniquet in an attempt to prevent epilation. As the oral route appeared to be as effective as the intravenous route, and as it was associated with a lower incidence of vomiting, it was adopted for this trial.

Nandrolone decanoate was also selected for this test series because the use of an injection avoids uncertainty about sublingual absorption and the injections of this compound need be no more frequent than once every 3 weeks. There is also less risk of jaundice than there is with testosterone (Sherlock, 1968).

### PATIENTS AND METHODS

*Selection of patients.*—Patients accepted for inclusion in the trial were those with histologically proven disease, with evidence of dissemination or post-irradiation recurrence not amenable to further surgery or radiotherapy. All patients had to be within 5 years of the menopause—either natural or induced by ovarian irradiation. No other cytotoxic or hormone therapy was permitted from the original referral to entry into the trial. On entry to the trial patients were randomly allocated to one of the 3 groups according to date of birth.

*Treatment schedules.*—Group C, whose date of birth fell between the 1st and 10th of the month, received cyclophosphamide alone in a dose of 50 mg thrice daily by mouth, the dose being modified at later visits according to the response and side-effects (especially marrow depression).

Group N, whose date of birth fell between the 11th and 20th of the month, received nandrolone decanoate 50 mg by intramuscular injection every 3 weeks.

Group C + N, whose date of birth fell between the 21st and 31st of the month, received both agents according to the same schedules as Groups C and N.

*Assessment of response.*—Response to treatment was assessed by change in visible, palpable or radiologically demonstrated deposits of tumour. A positive response was accepted if there was regression of more than 50% sustained for 3 months or more.

### RESULTS

Eighty-three patients were entered into the trial but 5 are excluded for the following reasons: Two in Group C + N died soon after starting treatment (at 3 days and at 2 weeks because of advanced disease); one in Group C + N and one in Group N were given a hormone preparation, prednisolone, before the trial drugs could be started; and one in Group C took the cyclophosphamide for 2–3 days, stopped it because she started an antibiotic and did not recommence it.

The number of patients in each group, and mean age at entry, are shown in Table II.

TABLE II.—*Mean Age at Entry*

| Group | Number | Age |
|---|---|---|
| C | 30 | 46·8±6·5 |
| N | 26 | 50·3±5·2 |
| C+N | 22 | 45·6±6·5 |
| Total | 78 | |

The 3 groups were studied for factors which might influence response to treatment but they seemed to be very similar. The stages at presentation and the proportions subjected to surgery or to radiotherapy as the initial management showed little difference between the groups. Local recurrence was the commonest indication for treatment and arose in 64 (86%) patients. The majority of patients with local recurrent disease had evidence of disease in other sites. There was no difference in the distribution of disease between groups, in particular the incidence of metastases in bone and liver.

### *Response*

The number of patients in whom a positive response was recorded is shown in Table III.

TABLE III.—*Number of Patients with Positive Objective Response with Mean Duration (Months)*

| Group | Number | Number of patients with response | Mean duration (months) |
|---|---|---|---|
| C | 30 | 7 | 6·2 |
| N | 26 | 0 | — |
| C+N | 22 | 1 | 8 |
| Totals | 78 | 8 | |

A positive response was recorded in 8 patients (10%), 7 of whom received cyclophosphamide alone. No patient who received nandrolone alone responded and only one patient who received the combination responded.

Eighteen additional patients showed an improvement which fell short of the criteria for a positive response and details are given in Table IV.

A comparison was made between patients in whom a response was noted

TABLE IV.—*Number of Patients with Partial Response*

| Group | Number | Response but under 50% tumour regression | Total, including responders from Table III |
|---|---|---|---|
| C | 30 | 6 | 13 |
| N | 26 | 4 | 4 |
| C+N | 22 | 8 | 9 |
| Totals | 78 | 18 | 26 |

and those who showed no improvement, to assess whether any particular feature could be identified that could indicate the possibility of a favourable response in future patients. The only positive feature noted was the higher proportion of patients with metastatic bone disease in those who did not respond to the therapeutic course.

Patients receiving cyclophosphamide alone had the longest mean period of survival (17·3 months), the survival period for the other 2 groups being similar (10 months). The mean survival for those patients with a positive response was 21 months compared with 11·7 months for those who did not ($P = 0{\cdot}10$).

### *Side-effects*

Adverse reactions were recorded in 31 (42%) patients. In 29 patients these were directly attributable to the drug and in the remaining two were a possibility. Details are given in Table V.

TABLE V.—*Incidence of Adverse Reactions*

| | Group | | |
|---|---|---|---|
| | C | N | C+N |
| Depression of white cell count below 3000/mm³ | 9 | 0 | 7 |
| Anaemia | 1 | 0 | 0 |
| Epilation | 7 | 0 | 8 |
| Vomiting | 6 | 0 | 3 |
| Haematuria | 1 | 0 | 0 |
| Tumour pain | 0 | 2 | 0 |
| Treatment stopped | 11 | 2 | 9 |

The number and nature of side-effects in those patients receiving cyclophosphamide and in those who received the combination were very similar. Five patients in the former group and 3 in the latter group were responding to treatment which had to be discontinued in view of depressed haemopoiesis.

### DISCUSSION

In this study cyclophosphamide proved to be the most effective agent, a positive response occurring in 23% of patients. Only one patient who received the combination of cyclophosphamide and nandrolone decanoate responded, and no response was noted in those receiving nandrolone alone.

Since nandrolone as a single agent is so ineffective in this menopausal group, it is not surprising that, in combination with cyclophosphamide, it adds nothing to the results achieved with cyclophosphamide alone. The unexpected finding in this trial is the relatively poor result obtained from the combination when compared with the use of cyclophosphamide alone. A possible explanation for this finding is that nandrolone interferes with the activation of cyclophosphamide. It now seems clear that cyclophosphamide is activated in the liver (Brock *et al.*, 1971; Brock, 1967; Foley, Friedman and Drolet, 1961) and there is evidence that prednisolone inhibits the activation of cyclophosphamide by rat liver (Hayakawa *et al.*, 1969). It is possible that nandrolone decanoate shares this property with prednisolone. However, the findings from this trial do not entirely support this hypothesis since if the cyclophosphamide is not being so freely activated in the group also receiving nandrolone, one would expect a further lowering of the incidence of adverse reactions, such as marrow depression and epilation, but this was not observed.

### *Conclusion*

Cyclophosphamide is more effective when used alone in these menopausal patients with advanced breast cancer than when combined with nandrolone decanoate.

### REFERENCES

BROCK, N. (1967) Pharmacologic Characterization of Cyclophosphamide (NSC 26271) and Cyclophosphamide Metabolites. *Cancer Chemother. Rep.*, **51**, 315.

BROCK, N., GROSS, R., HOHORST, J., KLEIN, H. O. & SCHNEIDER, B. (1971) Activation of Cyclophosphamide in Man and Animals. *Cancer, N.Y.*, **27**, 1512.

COLE, M. P. (1970) In *The Clinical Management of Advanced Breast Cancer*. Ed. C. A. F. Joslin and E. N. Gleave. Cardiff: Alpha-Omega Alpha Publishing Co. p. 43.

FOLEY, G. E., FRIEDMAN, O. M. & DROLET, B. P. (1961) Studies on the Mechanism of Action of Cytoxan. Evidence of Activation *in vivo* and *in vitro*. *Cancer Res.*, **21**, 57.

HAYAKAWA, T., KINNAI, N., YAMADA, R., KURODA, R., HIGASHI, H., MOGANI, M. & JINNAI. D. (1969) Effect of Steroid Hormone on Activation of Endoxan (Cyclophosphamide). *Biochem. Pharmac.*, **18**, 129.

SHERLOCK, S. (1968) *Diseases of the Liver and Biliary System*. London: Alden and Mowbury Ltd. p. 372.

WATSON, G. W. & TURNER, R. L. (1959) Breast Cancer; a New Approach to Therapy. *Br. med. J.*, i, 1315.